# Correction: Comprehensive proteomics and functional annotation of mouse brown adipose tissue

**DOI:** 10.1371/journal.pone.0244359

**Published:** 2020-12-17

**Authors:** The PLOS ONE Staff

The image for Fig 1 is incorrect. There is also an error in the caption for Fig 1. Please see the complete, correct Fig 1 and Fig 1 caption here. The publisher apologizes for the error.

**Fig 1:**
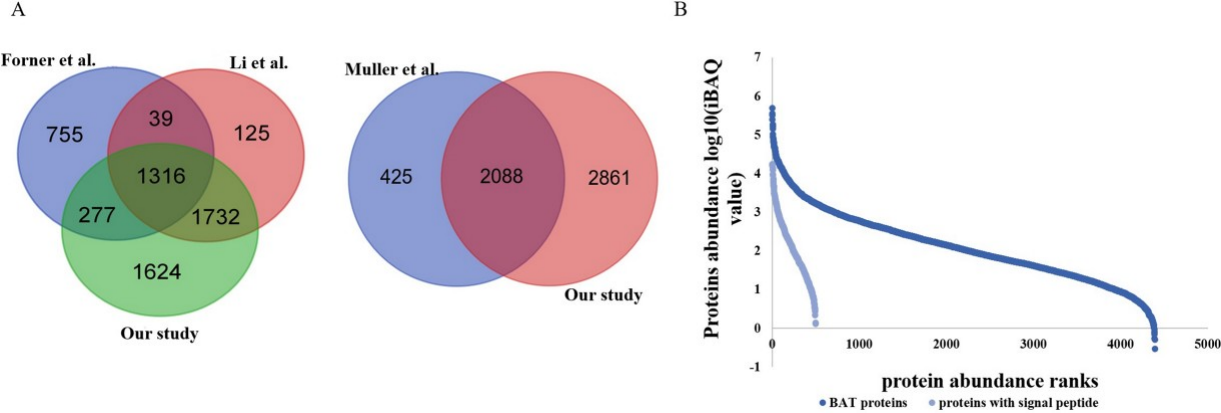
BAT proteome profile analysis. (A) A Venn diagram comparing BAT proteome large-scale studies. (B) The quantitative protein abundance ranges in BAT samples and the proteins that included a secreted signal peptide according to the iBAQ algorithm.
