# Supplementary figures and images for: Comprehensive proteomics and functional annotation of mouse brown adipose tissue
Source: PLoS One. 2020 May 6;15(5):e0232084. doi: 10.1371/journal.pone.0232084 (PMC7202602; doi:10.1371/journal.pone.0232084)

**S1. Fig The workflow of this study.**

**
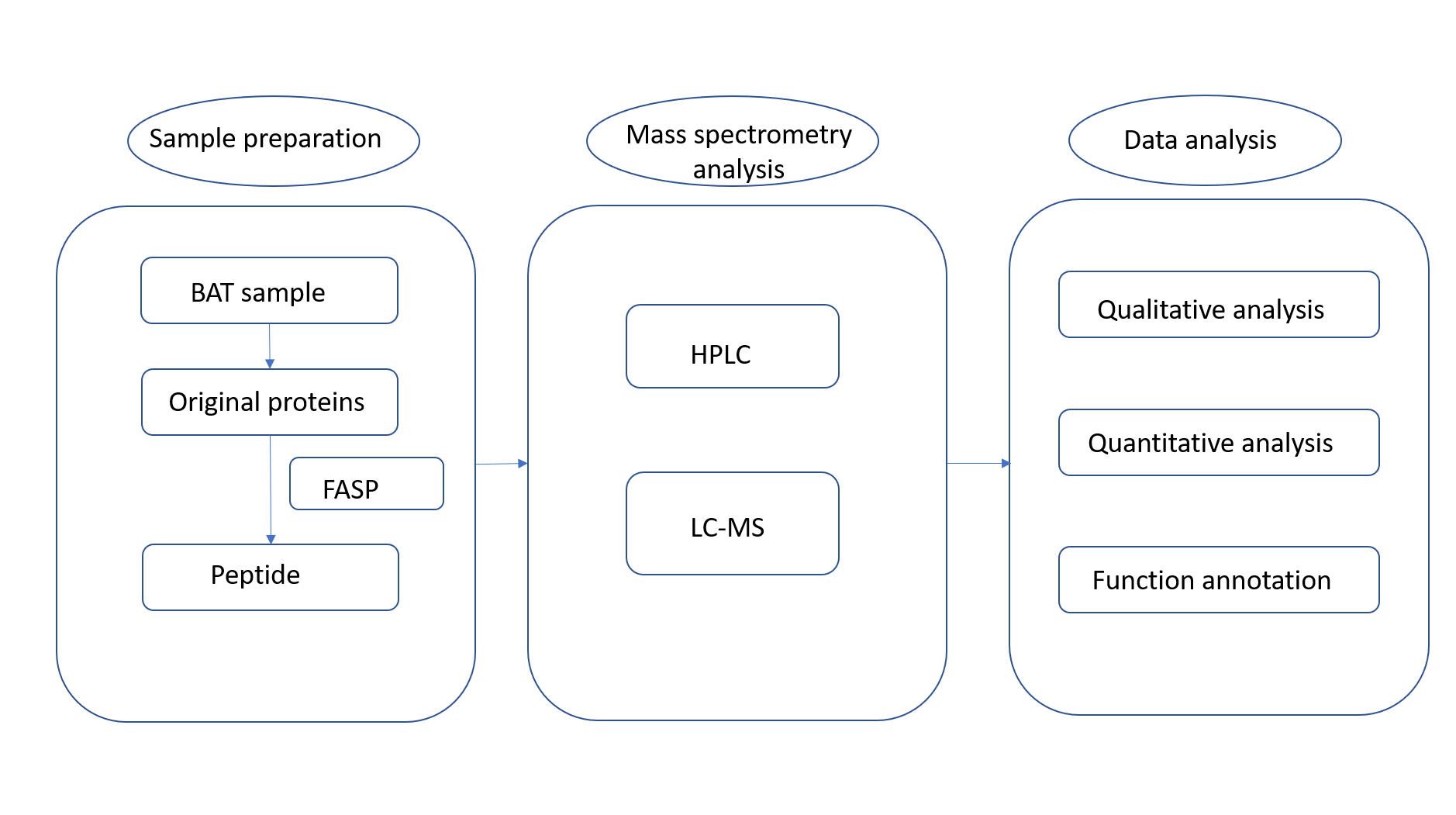
**

Supplement: S1 Fig — (DOCX) [file pone.0232084.s008.docx]

S3 Fig. The pathway analysis of BAT proteins and BAT PTM proteins


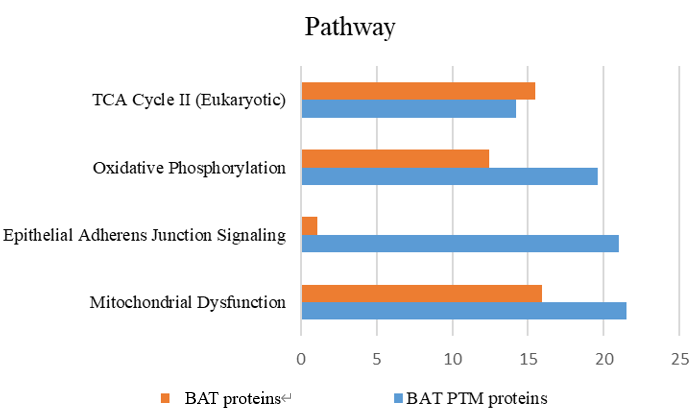

Supplement: S3 Fig — (DOCX) [file pone.0232084.s010.docx]
