# Supplementary material for: Comprehensive proteomics and functional annotation of mouse brown adipose tissue
Source: PLoS One. 2020 May 6;15(5):e0232084. doi: 10.1371/journal.pone.0232084 (PMC7202602; doi:10.1371/journal.pone.0232084)

**S2 Fig** **Repeatability of qualitative and quantitative experiments.** (A) the three runs of qualitative identified proteins. (B) the distribution diagram of CV of quantitative analysis. (C) Three repetitions of quantitative experiments

B

A

**169**

**142**

**118**

**4097**

**141**

**137**

**145**

**run1**

**run2**

**run3**

C


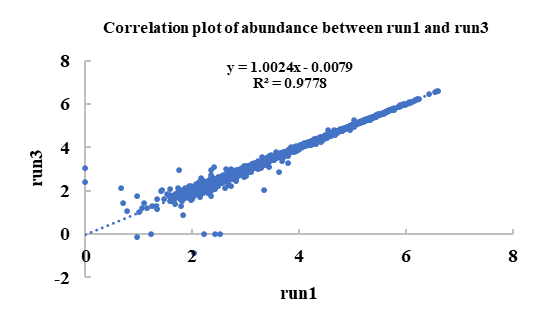

Supplement: S2 Fig — (DOCX) [file pone.0232084.s009.docx]
