# Supplementary material for: Comprehensive proteomics and functional annotation of mouse brown adipose tissue
Source: PLoS One. 2020 May 6;15(5):e0232084. doi: 10.1371/journal.pone.0232084 (PMC7202602; doi:10.1371/journal.pone.0232084)
Supplement: Attachment — Submitted filename: Response to Reviewers20200110.docx [file pone.0232084.s011.docx]

1. Although the current study suggested possible candidates for obesity markers, the authors should provide more information about those proteins. Under cold exposure, thermoneutrality, or HFD feeding, how is the level of the proteins changed?

Answer:

Thanks for the reviewer’s good advice. We agree your suggestions that it is important to analyze the BAT proteins under different environment, which is useful to illustrate the function of BAT.

This study is a pilot work and the main purpose is to profile the mouse brown adipose tissue proteome. And we also try to present the potential BAT function annotation and possible candidates for obesity markers by previous references analysis.

In the future, we will try to analyze the function and the change of these biomarkers under differential environment. We have added above statements in the manuscript (page 16 line 369-372).

And we revised the manuscript as follows:

According to previous studies the protein level of brown fat might be changed in different environments such as cold exposure, thermoneutrality, or HFD feeding.(Peres Valgas da Silva, Hernandez-Saavedra et al. 2019) (Small, Gong et al. 2018) (Kuipers, Held et al. 2019) Therefore, the BAT protein biomarker analysis under above conditions might be important to illustrate the function of brown fat, which should be investigated in the future.

2. Is there any sex or age difference? According to previous report (Choi, D.K., et al., Cell Physiol Biochem, 2011. 28(5): p. 933-48.), gender-dimorphic protein modulation in BAT may provide conclusive results showing higher expression of numerous proteins involved in thermogenesis and fat oxidation as well as lower expression of proteins contributing to fat synthesis in female rats than in male rats.

Answer:

Thank you for your suggestions. According to previous studies both sex and age could affect the BAT.

Studies of adult rats and mice indicate that females have a higher threshold for BAT activation (i.e. activate BAT sooner upon cooling than males). In human female BAT has a higher thermogenic capacity than male (Choi, Oh et al. 2011, Harshaw, Culligan et al. 2014)

For age, previous studies reported that both the activity and mass of BAT gradually decreases with the increase of age (Schosserer, Grillari et al. 2018). We have added above references in the manuscript (Line 102-103 on page 5).

According to previous studies(Choi, Oh et al. 2011, Harshaw, Culligan et al. 2014) (Schosserer, Grillari et al. 2018) Both gender and age can affect the expression level of BAT protein, so all the mice used in this experiment were C57BL/6 female mice with 6 weeks

3. It is interesting that high, middle, and low abundance proteins are involved in different signaling pathways. However, for signaling transduction, not only the level of proteins but also the post-translational modification of the proteins is important. It will be interesting and important to examine post-translational modifications of the representative signaling proteins in brown adipocytes under different environment.

Answer:

Thanks for the reviewer’s good advice on protein post-translational modifications, therefore, we added “post-translational modifications” section in revised manuscript as followings.

The MS/MS spectra were processed using Peaks Online (X build, 1.2.1010.68) to identify the post-translational modifications (PTM) of BAT proteome. Total 562 post-translational modifications proteins, 1665 peptides and 4464 MS/MS spectra were found in our data, including 142 acetylation, 117 phosphorylation and 179 methylation etc. (**S6 Table**). We also quantitatively analyzed the PTM proteins by label-free approach. The PTM proteins abundance accounted for 0.37% of total abundance. Among them methylation showed the highest abundance (0.07%).

To reveal the function of PTM proteins, they were input IPA for function annotation. The canonical pathways of PTM proteins were involved in mitochondrial dysfunction, epithelial adherens junction signaling and oxidative phosphorylation, which was consistent with BAT function (**Fig S3C)**. Among acetylation proteins, sirtuin 3(SIRT3) was an important protein in BAT function, which is the master deacetylase of deacetylation in the mitochondria. Previous study showed SIRT3 absence in mice resulted in impaired BAT lipid use, whole body thermoregulation, and respiration in BAT mitochondria without affecting UCP1 expression. Further analysis indicated SIRT3 controlled brown fat thermogenesis by deacetylation regulation of pathways upstream of UCP1(Sebaa, Johnson et al. 2019).

This study is a pilot work and the main purpose is to profile the mouse brown adipose tissue proteome, therefore we provided a PTM profile of BAT as you suggested. The representative signaling proteins in brown adipocytes under different environment were an important issue for BAT function analysis, which will be presented in future work.

we revised our study as follows: (Line 320-335 on page 14).

The MS/MS spectra were processed using Peaks Online (X build, 1.2.1010.68) to identify the post-translational modifications (PTM) of BAT proteome. Total 562 post-translational modifications proteins, 1665 peptides and 4464 MS/MS spectra were found in our data, including 142 acetylation, 117 phosphorylation and 179 methylation etc. (**S6 Table**). We also quantitatively analyzed the PTM proteins by label-free approach. The PTM proteins abundance accounted for 0.37% of total abundance. Among them methylation showed the highest abundance (0.07%).

To reveal the function of PTM proteins, they were input IPA for function annotation. The canonical pathways of PTM proteins were involved in mitochondrial dysfunction, epithelial adherens junction signaling and oxidative phosphorylation, which was consistent with BAT function (**Fig S3C)**. Among acetylation proteins, sirtuin 3(SIRT3) was an important protein in BAT function, which is the master deacetylase of deacetylation in the mitochondria. Previous study showed SIRT3 absence in mice resulted in impaired BAT lipid use, whole body thermoregulation, and respiration in BAT mitochondria without affecting UCP1 expression. Further analysis indicated SIRT3 controlled brown fat thermogenesis by deacetylation regulation of pathways upstream of UCP1. (Sebaa, Johnson et al. 2019).

| Name | Protein number | peptide number | #PSM | proportion |
| --- | --- | --- | --- | --- |
| Acetylation (K) | 142 | 338 | 1070 | 0.07% |
| Amidation | 217 | 302 | 547 | 0.04% |
| Methylation(KR) | 179 | 343 | 901 | 0.09% |
| Phosphorylation (STY) | 117 | 168 | 903 | 0.03% |
| Carboxylation (DKW) | 54 | 83 | 180 | 0.06% |
| Fluorination | 45 | 54 | 76 | 0.001% |
| Formylation (Protein N-term) | 16 | 20 | 47 | 0.003% |
| Formylation | 177 | 357 | 740 | 0.07% |
| sum |  |  |  | 0.37% |

Reviewer #2:

Minor recommendations: Please provide in the methods section the time of day that the tissue was collected in relation to the photoperiod that the mice were maintained on. There are clear differences in gene expression patterns and tissue function (e.g. glucose metabolism) according to the time of day as it related to diurnal and circadian rhythms (e.g. Van der Veen 2012; Zhang et al 2014; Zvonic et al 2006).

Answer:

Answer:

Thanks for your suggestions. We agree your comments that diurnal and circadian rhythms related to gene expression patterns and tissue function(Zvonic, Ptitsyn et al. 2006)

We have added above information in the manuscript: (Line 95-100 on page 5).

“According to previous studies diurnal and circadian rhythms related to gene expression patterns and tissue function(Zvonic, Ptitsyn et al. 2006, van der Veen, Shao et al. 2012, Zhang, Lahens et al. 2014). Female mice were housed at room temperature in a 12:12-h light-dark cycle at 23±2°C ………And the interscapular BATs were excised directly in 9 a.m by tweezers and scissors from the lateral scapular area of the mice back”

Reviewer #3:

Major Concerns:

- title should include protein or proteomics so as to better match the content of the article

Answer:

Thanks for your suggestions, the title had been revised as below:

“Comprehensive proteomic dataset and functional annotation of mouse brown adipose tissue“

- female mice are included in the study, is there reason to anticipate sex differences?

Answer:

Thank you for your suggestions. According to previous studies sex could affect the BAT. Studies of adult rats and mice indicate that females have a higher threshold for BAT activation (i.e. activate BAT sooner upon cooling than males). In human female BAT has a higher thermogenic capacity than male (Choi, Oh et al. 2011, Harshaw, Culligan et al. 2014)

We have added above information in the manuscript (Line 102-103 on page 5).

“According to previous studies(Choi, Oh et al. 2011, Harshaw, Culligan et al. 2014) (Schosserer, Grillari et al. 2018) Both gender and age can affect the expression level of BAT protein, so all the mice used in this experiment were C57BL/6 female mice with 6 weeks.”

- Ref 11 may be misinterpreted in the Introduction – BAT secretes factors that act in an autocrine/paracrine manner on BAT itself to carry out these functions?? More updated papers on BATokines should be cited, since recent work has investigated BAT secreted factors and their roles in energy metabolism.

Answer: Thank you for your comments. According to your suggestion, we have revised the manuscript as followings (Line 48-51 on page 3)

BAT is a secretory tissue, which can produce BATokines. BATokines act locally to control the remodeling of BAT, and they also act on peripheral organs, including the liver, skeletal muscle, pancreas, bone, the immune system and the CNS, thus affect systemic glucose levels in a thermogenesis-dependent and -independent manner.(Klepac, Georgiadi et al. 2019)

1. How does the current mass spec data set compare to the set produced in Ref 22 by Li et al? (and any other similar mass spec BAT proteomics studies?)

Answer: Thanks for your comments.

The protein accession numbers of previous report and our data were transferred to gene entry names using Uniprot website( <https://www.uniprot.org/>), then we make a qualitative comparison at the gene level.

2. Are there technical considerations (and what do they mean – clarification on Table 1 would help), tissue collection protocols, mouse strain/age/sex that may create different protein data sets? Fig 2 is helpful but does not include these parameters, and the 3 studies outlined have relatively little overlap with 1316 proteins in common.

Answer: Thank you for your suggestions.

According to previous studies, the protein identification will be different because of different tissue collection protocols, mouse strain/age/sex (Choi, Oh et al. 2011, Harshaw, Culligan et al. 2014) (Schosserer, Grillari et al. 2018) (Zvonic, Ptitsyn et al. 2006, van der Veen, Shao et al. 2012, Zhang, Lahens et al. 2014) . Besides above factors, many technical factors also influence the results, including protein digestion method, peptide separation method, mass spectrometry and database search software. Therefore, above factors lead to the relatively low overlap between different studies.

To clearly illustrate the results, according to your suggestions, we have added above factor in Table 1.

Have any studies been done in human WAT or BAT (or beige, the most common brown adipocyte in humans)?

Answer: Thank you for your suggestions

In 2016, Muller et al. present comprehensive proteome analysis of human brown and white adipose tissues. 2519 adipocyte proteins were identified (Muller, Balaz et al. 2016) and 2088 were found in our study.

we added the comparison with human BAT in the manuscript (Line 201-202 on page 8).

“In addition, we compared the proteins we identified to human adipose tissue proteins, the result showed that 2088 proteins were overlapped with Muller et al.(Muller, Balaz et al. 2016)

|  | **Year** | **Mouse**  **strain/age/sex** | **Number of identifications** | **Fractionation method** | **MS instrument** | **Identification quality (FDR)** | **Reference** |
| --- | --- | --- | --- | --- | --- | --- | --- |
| 1 | 2001 | 8 week old C57Bl/6J  females | 37 | 2-DE | Q-TOF-MS | N/A | Sanchez et al.(Sanchez, Chiappe et al. 2001) |
| 2 | 2004 | C57BL/6 female mice with 6 weeks | 20 | 2-DE | Q-TOF-MS | N/A | Schmid et al.(Schmid, Converset et al. 2004) |
| 3 | 2007 | Wistar male rats | 58 | 2-DE | UltraFlex II MALDI-TOF-TOF | N/A | Navet et al.(Navet, Mathy et al. 2007) |
| 4 | 2009 | C57BL/6 mice (5 to 10 weeks old) | 2434 | SILAC | LTQ-Orbitrap | Protein level< 1% | Forner et al.(Forner, Kumar et al. 2009) |
| 5 | 2011 | Male and female SLC Sprague-Dawley (SD) rats (5 weeks  of age) | 55 | 2-DE | MALDI-TOF-MS | N/A | Choi et al.(Choi, Oh et al. 2011) |
| 6 | 2015 | C57BL/6 female mice with 6 weeks | 3048 | iTRAQ | Triple TOF 5600 | Protein level< 1% | Li et al.(Li, Zhao et al. 2015) |
| 7 | 2016 | Wild-caught thirteen-lined ground squirrels | 778 | iTRAQ | Velos  Orbitrap | Protein level< 1% | Ballinger et al.(Ballinger, Hess et al. 2016) |
| 8 | 2016 | 11 patients | 2019 | TMT | Orbitrap Fusion tribrid | Protein level< 1% | Muller etal.(Muller, Balaz et al. 2016) |
| 9 | 2019 | C57BL/6 female mice with 6 weeks | 4949 | RPLC | Triple TOF5600 | Protein level<1% | This study |

- 6 BAT samples were collected (was overlying WAT carefully removed??) and pooled – how does this affect statistical rigor to have no biological replicates?

Answer: Thank you for the reviewers’ suggestions.

Based on our observations and previous study reports(Vargas-Castillo, Fuentes-Romero et al. 2017), the BAT and WAT can be clearly distinguished by appearance, since BAT has a characteristic yellow-brown color due to its high content of [mitochondria](https://www.sciencedirect.com/topics/medicine-and-dentistry/mitochondrion) and the color of white adipose tissue is white ,thus we took out the BAT directly from the back of the mouse and removed the white fat by color.

In proteomic database report several samples were usually pooled into one sample to reduce the individual effect on the final results. Then a comprehensive analysis will be done on the pooled sample. This is a normal strategy for proteomic database analysis(Vargas-Castillo, Fuentes-Romero et al. 2017). In this study we also adapted above strategy.

- Were some proteins potentially from non-adipocytes? (ie: BAT cotains stem cells, immune cells, blood/neural tissue, etc. aside from pre-adipocytes and adipocytes). In the top 10 proteins, serum albumin, hemoglobins, and potentially other proteins are part of contaminating blood likely...

Answer: Thanks for your question. previous studies reported that brown adipose tissue contained the non-adipocytes including stem cells, (Villarroya, Cereijo et al. 2019)immune cells, blood/neural tissue,(Payab, Goodarzi et al. 2018)stromal-vascular (S-V) cells (non-adipocytes)(Song, Fukui et al. 2010), Therefore, our database included the proteins from these non-adipocytes. In addition, blood vessels are also around brown adipose tissue, thus the proteins from plasma were also included in our database.

We have added your suggestions in the manuscript: (Line 191-195 on page 8)

Previous studies reported that brown adipose tissue contained the non-adipocytes including stem cells, (Villarroya, Cereijo et al. 2019)immune cells, blood/neural tissue,(Payab, Goodarzi et al. 2018)stromal-vascular (S-V) cells (non-adipocytes)(Song, Fukui et al. 2010), Therefore, our database included the proteins from these non-adipocytes. In addition, blood vessels are also around brown adipose tissue, thus the proteins from plasma were also included in our database.

- Since numerous mitochondrial proteomic studies in BAT have been undertaken, which of these proteins are thought to be mitochondrial (ie: in mitochondrial function and/or mitochondrially encoded)?

Answer: Thank you for your suggestions, according to your suggestions, we add the related results about BAT mitochondrial proteins as follows (page 14 line307-319)

We used GO analysis to annotate the cellular components of all the proteins, and 214 mitochondria proteins were found. These BAT mitochondrial proteins were subdivided into three groups according to their abundance, 72 high-abundance, 90 middle-abundance, 39 low-abundance proteins.

The functions of BAT proteins were mainly related to the regulation of glucose and lipid metabolism, protein synthesis and apoptosis. The mitochondrial proteins were mainly involved in pathways of valine degradation I, isoleucine degradation I, ketogenesis, sirtuin signaling pathway and tRNA charging, which were related to oxidative phosphorylation and amino acid metabolism (**Fig4A**).

The high-abundance proteins were mainly involved in amino acid degradation, the middle-abundance proteins were mainly related to amino acid biosynthesis and degradation, and the low-abundance proteins were found to be involved in apoptotic signaling pathways such as death receptor Signaling and thioredoxin Pathway. (**Fig4B**)

pathway

|  | Mitochondrial proteins | BAT proteins |
| --- | --- | --- |
| Valine Degradation I | 9.33 | 7 |
| Isoleucine Degradation I | 8.37 | 5.92 |
| Ketogenesis | 7.46 | 2.69 |
| Leucine Degradation I | 5.79 | 3.12 |
| Mevalonate Pathway I | 5.2 | 4.78 |
| tRNA Charging | 6.81 | 22.2 |
| Mitochondrial Dysfunction | 5.84 | 33 |
| Sirtuin Signaling Pathway | 7.36 | 23.7 |
| Protein Ubiquitination Pathway | 0.801 | 25.5 |
| Oxidative Phosphorylation | 3.11 | 24.1 |
| mTOR Signaling | 0 | 22.9 |
| EIF2 Signaling | 0 | 35.3 |
| Regulation of eIF4 and p70S6K Signaling | 0 | 29.4 |

pathway

|  | high abundance mitochondrial proteins | middle abundance mitochondrial proteins | low abundance mitochondrial proteins |
| --- | --- | --- | --- |
| Valine Degradation I | 12.6 | 0 | 0 |
| Isoleucine Degradation I | 11.1 | 0 | 0 |
| Leucine Degradation I | 7.61 | 0 | 0 |
| Ketogenesis | 7.39 | 0 | 0 |
| Sirtuin Signaling Pathway | 7.14 | 0 | 0 |
| tRNA Charging | 0 | 6.03 | 0 |
| L-carnitine Biosynthesis | 0 | 1.85 | 0 |
| Proline Degradation | 0 | 1.85 | 0 |
| Glutamate Degradation II | 0 | 1.85 | 0 |
| Aspartate Biosynthesis | 0 | 1.85 | 0 |
| tRNA Charging | 0 | 0 | 2.6 |
| L-glutamine Biosynthesis II (tRNA-dependent) | 0 | 0 | 2.41 |
| Induction of Apoptosis by HIV1 | 0 | 0 | 2.21 |
| Death Receptor Signaling | 0 | 0 | 1.88 |
| Thioredoxin Pathway | 0 | 0 | 1.86 |

- Is PANTHER protein classification and annotation with GO terms enough for a proteomics data set? How about pathway analysis in terms of protein interactions and protein networks?

Answer: Thank you for your suggestions

Gene Ontology (GO) project provides the annotation of data. The GO analysis including three parts: cellular component, molecular function, biological process. From these three aspects of annotation, we can learn about the distribution of proteins in cells, the functions and biological processes of our proteins.

But GO analysis only provides an annotation to give us an initial understanding of the proteins in the data, and more accurate analysis of the protein data is mainly through Ingenuity Pathway Analysis (IPA) software.

IPA is based on the database of various biomedical literature and integrated from third-party databases. In addition, IPA use a suite of algorithms and tools for inferring and scoring regulator networks upstream and downstream of gene-expression data based this database. Furthermore, it predicted the related pathway analysis and diseases and function analysis (Kramer, Green et al. 2014). Currently, IPA analysis is a commonly used proteomic analysis. (Wei, Meng et al. 2019). Therefore, we used both GO annotation and IPA analysis in our study.

Minor Concerns:

- Fig 1 is a bit vague and not very useful – adding specific details would help the reader orient to the study and analysis of data

Answer: Thanks for your suggestion, we adjusted the definition of Fig 1 and moved this figure to the S1 Fig.

- Fig. 3 -4 can not be read – please increase size of Figures and text. I can’t see any words at all even when I zoom in, so I can not even comment on these data.

Answer: Thanks for your suggestion, I am sorry about the figure that you cannot read it. We recalibrated the picture definition
