# Supplementary material for: Comprehensive proteomics and functional annotation of mouse brown adipose tissue
Source: PLoS One. 2020 May 6;15(5):e0232084. doi: 10.1371/journal.pone.0232084 (PMC7202602; doi:10.1371/journal.pone.0232084)
Supplement: Attachment — Submitted filename: Response to Reviewers.docx [file pone.0232084.s012.docx]

Reviewer #2: The requested information as stated in the methods section is still insufficient or incorrect. Please state the time of lights OFF and time of lights ON, and time of collection of tissue within the methods section. The authors citation of Van der Veen 2012 (citation 26) is incorrect, please use the correct citation: Van der Veen (2012) A diurnal rhythm in glucose uptake in brown adipose tissue revealed by in vivo PET-FDG imaging. van der Veen DR, Shao J, Chapman S, Leevy WM, Duffield GE. Obesity (Silver Spring). 2012 Jul;20(7):1527-9. doi: 10.1038/oby.2012.78. Epub 2012 Mar 26. PMID: 22447290.

Answer:

Thanks for the reviewer’s good advice.

We revised the manuscript as follows:

“Female mice were housed at room temperature in a 12:12-h light-dark cycle at 23±2°C, with free access to water and diet. The time of lights on is eight a.m., the time of lights off is eight p.m., and the time of collection tissue is nine a.m.”

In addition, we have modified reference 26. Thank you very much for your correction.
